# Sequential EMT-MET induces neuronal conversion through Sox2

**DOI:** 10.1038/celldisc.2017.17

**Published:** 2017-05-30

**Authors:** Songwei He, Jinlong Chen, Yixin Zhang, Mengdan Zhang, Xiao Yang, Yuan Li, Hao Sun, Lilong Lin, Ke Fan, Lining Liang, Chengqian Feng, Fuhui Wang, Xiao Zhang, Yiping Guo, Duanqing Pei, Hui Zheng

**Affiliations:** 1 CAS Key Laboratory of Regenerative Biology, Joint School of Life Sciences, Guangzhou Institutes of Biomedicine and Health, Chinese Academy of Sciences, Guangzhou Medical University, Guangzhou, China; 2 University of Chinese Academy of Sciences, Beijing, China; 3 Guangdong Provincial Key Laboratory of Stem Cell and Regenerative Medicine, Guangzhou, China

**Keywords:** TuJ^+^ cells, sequential EMT-MET, proliferation, Sox2

## Abstract

Direct neuronal conversion can be achieved with combinations of small-molecule compounds and growth factors. Here, by studying the first or induction phase of the neuronal conversion induced by defined 5C medium, we show that the Sox2-mediated switch from early epithelial–mesenchymal transition (EMT) to late mesenchymal–epithelial transition (MET) within a high proliferation context is essential and sufficient for the conversion from mouse embryonic fibroblasts (MEFs) to TuJ^+^ cells. At the early stage, insulin and basic fibroblast growth factor (bFGF)-induced cell proliferation, early EMT, the up-regulation of *Stat3* and *Sox2*, and the subsequent activation of neuron projection. Up-regulated *Sox2* then induced MET and directed cells towards a neuronal fate at the late stage. Inhibiting either stage of this sequential EMT-MET impaired the conversion. In addition, Sox2 could replace sequential EMT-MET to induce a similar conversion within a high proliferation context, and its functions were confirmed with other neuronal conversion protocols and MEFs reprogramming. Therefore, the critical roles of the sequential EMT-MET were implicated in direct cell fate conversion in addition to reprogramming, embryonic development and cancer progression.

## Introduction

Neural stem cells (NSCs) and neurons induced directly from somatic cells have the potential to treat neurodegenerative diseases, such as Alzheimer’s disease and Parkinson’s disease [1, 2]. Methods that directly induce NSCs have been optimized from using the four Yamanaka factors, *Oct4*, *Klf4*, *c-Myc* and *Sox2*, to using only small-molecule compounds and a hypoxic environment [3, 4]. Protocols to induce functional neurons have evolved from using combinations of transcription factors, including Ascl1, Brn2 and Myt1l, to using a single transcription factor, neuronal differentiation 1 (NeuroD1) [5, 6]. In 2015, five reports from four laboratories, including ours, induced neurons or neuron-like cells *in vitro* and *in vivo* using only small-molecule compounds and growth factors, both from mouse and human somatic cells [7–11].

The reported neuronal conversions all included two phases and used two mediums, the initial induction medium in the induction phase and the late maturation medium in the maturation phase [8, 9, 11]. The initial induction medium induced somatic cells towards neuron-like or TuJ^+^ cells, and the late maturation medium further converted TuJ^+^ cells to functional neurons. Because maturation medium alone cannot induce TuJ^+^ cells, initial induction medium is critical to induce neuronal characteristics during the conversion although it cannot fully generate functional neurons. In addition, the major differences among these five protocols lie in the small-molecule compounds used in the induction phase, although valproic acid (VPA, histone deacetylase inhibitor), CHIR99021 (glycogen synthase kinase 3 inhibitor) and forskolin/cAMP (cAMP inducer) have been used in at least three protocols [7–11]. Thus the mechanisms underlying the initial induction phase were focused in the current investigations.

In our previous report, neuronal characteristics can be induced with simple defined 5C medium, which only includes DMEM/F12, N2, bFGF, leukemia inhibitory factor, vitamin C and 2-mercaptoethanol [11]. Based on the morphological and gene expression changes during the conversion with 5C medium [11], we propose a sequential epithelial–mesenchymal transition (EMT)-mesenchymal–epithelial transition (MET), which has been reported during embryonic development, cancer progression and the generation of induced pluripotent stem cells (iPSCs) [12,13, 14]. We hypothesized that the early EMT may poise the cells in a state more suitable for further cell fate conversion [15, 16]. This hypothesis was first tested during the 5C-induced conversion and then during the conversions with other protocols.

## Results

### Facilitated proliferation and migration during the conversion

5C medium converts mouse embryonic fibroblasts (MEFs) into neuron-like cells or TuJ^+^-positive cells within 14 days. However, these neuron-like cells or TuJ^+^-positive cells are not fully functional neurons [11]. These neuron-like cells can be further converted to neurons by using maturation medium. The other reported protocols which use small-molecule compounds to induce direct neuronal conversions also include at least two phases [7,8,9, 10], the earlier induction phase and the later maturation phase. The induction medium converts the cell fate of MEFs to neuronal cell fate, while the maturation medium further converts the neuron-like or intermediate cells to functional neurons. As maturation medium cannot induce neuronal conversion alone, it is reasonable to suggest that the essential role of induction medium in inducing neuronal characteristics. In the current study, the mechanisms employed by the induction medium, or current 5C medium, to induce neuronal characteristics were investigated.

The expression of markers of fibroblasts, MEFs, primary astrocytes, neurons and NSCs were determined by quantitative PCR (qPCR) in TuJ^+^ cells and remaining cells. Based on the gene expression listed in Supplementary Figure S1A and B, the current neuron-like cells were closer to primary neurons. As the fibroblast markers, *Snai1/2* and *Twist2*, have significant higher expression in MEFs than in primary astrocytes, while *Gfap* and *Slc1a3* are more specific for astrocytes [17], the remaining cells were closer to MEFs (Supplementary Figure S1C). Both kinds of cells were far away from primary astrocytes or NSCs.

**Figure 1:**
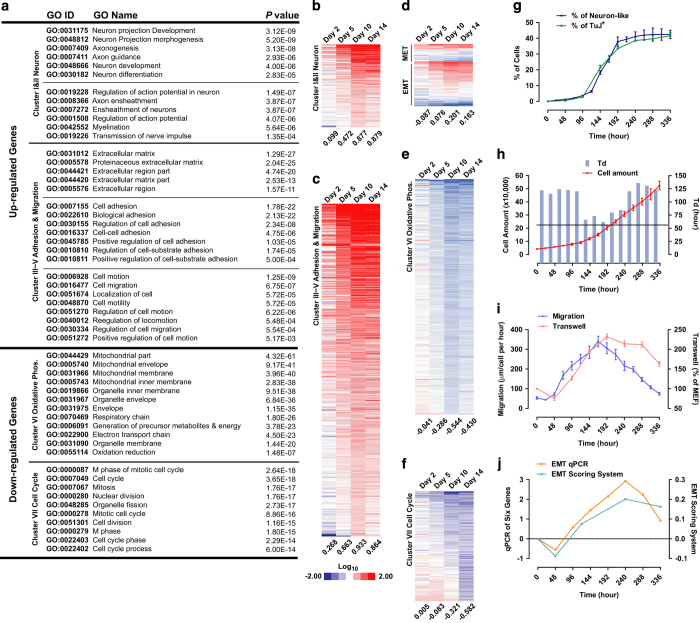
Cell proliferation and migration are induced during the conversion. (**a**–**f**) RNA-seq was performed during 5C-induced neuronal conversion. Genes with significant expression changes (over twofold) were enriched in seven gene ontology (GO) clusters (**a**). Heatmaps were used to summarize the expression changes of genes in these seven clusters (**b**–**f**). Averages of the log_2_ values (EMT scores in **d**) are provided below the heatmaps. (**g**–**j**) Percentages of neuron-like and TuJ^+^ cells were determined by cell morphology and immunofluorescence, respectively, and were consistent with each other (**g**). Cell amounts and doubling times are plotted in **h**. Distances that cells migrated and transwell results are summarized in **i**. The EMT scores based on RNA-seq and qPCR are listed in **j**.

Defined 5C medium (Supplementary Table S1) was used to treat MEF cells for 14 days, and gene expression profiles were analysed on days 0, 2, 5, 10, and 14 [11]. Seven clusters of enriched gene ontology terms were identified for the genes whose expression changed significantly (Figure 1a and Supplementary Table S2). Consistent with the gradual acquisition of neuronal characteristics, genes related to neuron projection and neuron cell fate (Clusters I and II) were up-regulated (Figure 1b). The expression changes of genes in Cluster III–V that related to adhesion and migration suggested a sequential EMT-MET (Figure 1c), which was further confirmed by the expression changes of EMT and MET markers (Figure 1d). Suppression of genes related to oxidative phosphorylation (Cluster VI) and cell cycle (Cluster VII) suggested a metabolic switch and proliferation regulation (Figure 1e and f).

To further establish the connection between proliferation/migration with neuronal conversion, a live-cell imaging system was then used (Supplementary Movies S1, S2, S3). Two criteria—length-to-width ratio over 5 and at least two neurite outgrowths—were used to identify neuron-like cells and resulted in a significant overlap (over 85%) with TuJ^+^ cells (Figure 1g). A significant increase of neuron-like or TuJ^+^ cells was observed between days 4 and 9, which correlated with the higher proliferation rate (Figure 1h), enhanced cell migration (Figure 1i) and EMT at the early stage (Figure 1j, Supplementary Tables S3 and S4), which suggested the beneficial roles of enhanced cell proliferation and migration for the neuronal conversion.

However, a small decrease in cell migration was observed on day 2 during 5C-induced conversion (Figure 1g–j). Such decrease was attributed to the serum removal when 5C medium was used to replace original fetal bovine serum (FBS) medium, as pre-treatment of serum-free medium inhibited proliferation partially by increasing apoptosis, promoted cells towards an epithelial state, impaired cell migration and prohibited neuronal induction by 5C medium (Supplementary Figure S1D–H).

Although enhanced proliferation and migration correlated with the increase of TuJ^+^ cells at the early stage (days 4–9), the proliferation inhibition and migration arrest at the late stage (days 10–14) were consistent with the characteristics of mature neurons. In addition, although a constant change towards primary neurons was suggested during the current conversion (Figure 1b, Supplementary Figure S2A and B), the expression changes for genes related to EMT/MET, oxidative phosphorylation (OX) and glycolysis (Gly) suggested that the late stage is more similar to neuronal conversion than the early stage (Supplementary Figure S2C–F and Supplementary Table S5). Furthermore, *Bcl2*, which facilitates the metabolic switch from glycolytic to oxidative metabolism during neuronal conversion with Ascl1 [18], was suppressed at the early stage and up-regulated at the late stage (Supplementary Figure S2F). Thus, the early stage of the current conversion is either redundant or is to prepare cells for further conversion.

### Early mitosis is critical for the conversion

**Figure 2:**
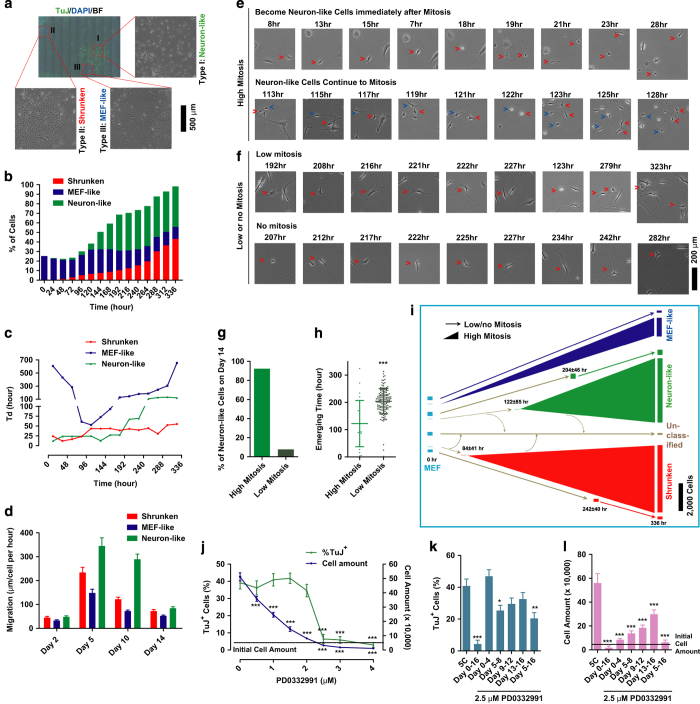
Induction of neuron-like cells requires mitosis. (**a**–**d**) The majority (over 97%) of cells on day 14 were classified as neuron-like, shrunken and MEF-like cells (**a**). The percentages (**b**), doubling times (**c**) and migration abilities (**d**) of these three types of cells are summarized. (**e**, **f**) Neuron-like cells could be induced with high or low (direct conversion) mitosis. In the first picture of each line of pictures, the cells selected for tracing were marked with red arrows. These cells and their daughter cells were marked in the following pictures. When two cells were traced, red and blue arrows were used to distinguish them. (**g**, **h**) The percentages of neuron-like cells induced with high or low mitosis are summarized after tracing 500 randomly selected MEFs (**g**). The averaged emerging time of these two kinds of cells are in **h**. (**i**) Summary of the 5C-induced conversion with 500 randomly selected MEFs. The averaged emerging time of different kinds of cells is also provided. (**j**–**l**) The concentration-dependent and time-dependent effects of PD0332991 on proliferation and TuJ^+^ cell induction.

By using the live-cell imaging system, each individual cell on day 14 during the conversion was traced back to MEFs, on day 0. Actually, three major types of cells, neuron-like, shrunken and MEF-like cells, accounted for more than 97% of the surviving cells on day 14 (Figure 2a and b). MEF-like cells accounted for about 25% cells on day 0 but only about 10% cells on day 14, because of their low proliferation rate (Figure 2b and c). Neuron-like and shrunken cells were not observed at the beginning of the conversion but account for ~90% cells on day 14, because of the conversion from MEFs and high proliferation rates (Figure 2b and c). The migration of these three types of cells all peaked around days 5–10, which correlated with their higher proliferation in the similar time period (Figure 2c and d).

The generation of neuron-like cells was then traced with live-cell imaging. Some cells acquired neuron-like morphology immediately after one particular round of mitosis and continued to produce more neuron-like cells (Figure 2e). However, some other cells acquired neuron-like morphology with low or even no mitosis (Figure 2f). Although the number of converted cells with low mitosis was ~6-fold higher than that of cells that converted with high mitosis, their contribution to the final neuron-like cells on day 14 was only ~8% (Figure 2g and h). In addition, the conversions of cells with low mitosis were much later than the conversion of cells with high mitosis. Thus, mitosis is critical for the induction of neuron-like cells from MEFs.

Similar phenomena were observed with shrunken and MEF-like cells. More cells were converted with low mitosis than with high mitosis, but their contributions to the final cells were less and averaged conversion time was later (Supplementary Figure S3A–F). Therefore, mitosis is essential for the conversion of all three types of cells.

As summarized in Figure 2i, ~53% of initial MEFs converted to neuron-like and shrunken cells either with high mitosis at the early-to-middle stage, or converted directly at the middle-to-late stage without significant mitosis. Approximately 23% and 24% of initial MEFs converted to MEF-like cells and unclassified cells, respectively. However, as MEF-like and unclassified cells suffered from the initial proliferation inhibition resulting from serum removal, and more cells underwent apoptosis during the conversion, the final percentages of MEF-like and unclassified cells converted from MEFs on day 14 were small (Supplementary Figure S3G–I).

To further confirm the contribution of cell proliferation to the conversion, the regulatory effects of the highly selective CDK4/6 inhibitor, PD0332991 [19], on conversion were then determined. PD0332991 did not significantly affect the final percentage of TuJ^+^ cells until concentrations of 2.5 μm or higher were used, which fully blocked proliferation (Figure 2j). In addition, PD0332991 had a greater inhibitory effect on the final number of TuJ^+^ cells when used at the early stage and a greater inhibitory effect on the final percentage of TuJ^+^ cells when used at the late stage (Figure 2k and l), possibly because of the different time-dependent regulations on the proliferation of the three cell types (Figure 2c).

If cell proliferation is important, the initial cell density and activation of Notch pathway should also be important for the conversion. Actually, Notch pathway activation was observed during the conversion, especially with a high initial density of MEFs (Supplementary Figure S4A and B). Using DAPT, a γ-secretase inhibitor, to block the Notch pathway increased both the number and percentage of final TuJ^+^ cells, which was consistent with the Notch pathway activator Jagged1 (Supplementary Figure S4C and D). DAPT increased the percentage of TuJ^+^ cells by supporting the further proliferation of TuJ^+^ cells and suppressing the proliferation of other cells at the late stage (Supplementary Figure S4E and F). The optimal density of initial MEFs was between 20 000 and 50 000 per well in a 6-well plate (Supplementary Figure S4G).

### Sequential EMT-MET induced by insulin and bFGF

**Figure 3:**
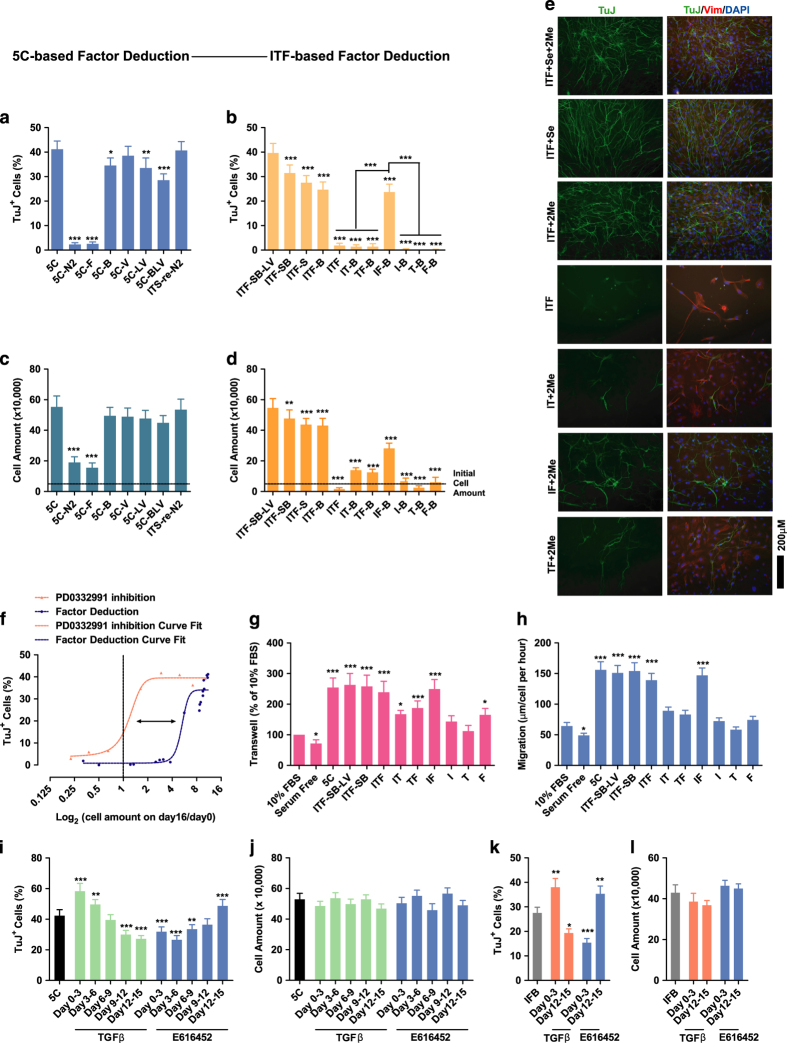
Sequential EMT-MET is essential for the induction. (**a**–**e**) The abilities of 5C and modified mediums to induce proliferation (**a**, **c**) and TuJ^+^ cells (**b**, **d**) were determined with FACS on day 16. Representative immunofluorescence images are provided to indicate the essential roles of insulin and bFGF (**e**). (**f**) Final cell amounts on day 16 were plotted against the percentages of TuJ^+^ cells based on the results from PD0332991 (Figure 2j–l) and factor deduction (**a**–**d**). The shift between the two curves is indicated with a black arrow. (**g**, **h**) Transwell results and migration distances were used to determine cell migration induced by 5C and other modified mediums. (**i**–**l**) TGFβ and E616452 were used to induce EMT and MET, respectively, during the conversion induced by 5C or IFB medium. Proliferation rates and the number of TuJ^+^ cells were determined on day 16. I, T, F, S, B (2me), L, and V was used to suggest that insulin, transferrin, bFGF, sodium selenite, 2-mercaptoethanol, LIF, and vitamin C, respectively, was supplemented in the basal DMEM/F12 (1:1) medium.

We next investigated which gradient or gradients in 5C medium accounted for the stimulated proliferation. Separately purchased and mixed insulin (I), transferrin (T) and sodium selenite (S) were used to replace N2, and no difference in proliferation or conversion was observed (Figure 3a and b). Furthermore, with sodium selenite or 2Me (B) supplemented in the medium, both insulin and bFGF were found to be essential for proliferation and TuJ^+^ cell induction (Figure 3a–e). Whether insulin and bFGF induced a similar conversion in the mouse brain was then determined. As indicated in Supplementary Figures S6, 5C medium and IFB (DMEM/F12 supplemented with insulin, bFGF and 2-me) medium induced better recovery, as demonstrated with increased tissue inside the lesion that resulted from medium infusion, weaker immunofluorescence response of glial fibrillary acidic protein (GFAP) and more EdU^+^ cells inside and surrounding the lesion.

When the final cell amounts and percentages of TuJ^+^ cells from samples tested during factor deduction were plotted and compared to the curve generated with PD0332991, a curve shift was identified (Figures 2j and 3a–f), which suggested that insulin and bFGF contribute to TuJ^+^ cell induction via other pathways in addition to promoting proliferation. The first hypothesis was sequential EMT-MET because it also facilitates iPSC generation [12].

Both insulin and bFGF increased cell migration as demonstrated using transwell assays, wound-healing assays and live-cell tracing (Figure 3g and h and Supplementary Figure S5). In addition, as demonstrated by the expression of EMT or MET markers, insulin and bFGF were necessary and sufficient to induce sequential EMT-MET during the conversion (Supplementary Table S6). TGFβ and E616452 (also named Repsox) were then used to modulate the sequential EMT-MET (Supplementary Table S6). Inhibiting either early EMT or late MET impaired the conversion (Figure 3i and j). Similar regulatory roles of TGFβ and E616452 were observed with neuronal conversion induced by IFB medium (Figure 3k and l). Therefore, insulin- and bFGF-induced sequential EMT-MET is also required for the induction of TuJ^+^ cells.

### Sox2 can replace sequential EMT-MET

To establish the connection between sequential EMT-MET and the neuronal conversion, Pscan [20] was used to identify potential transcription factors that are directly upstream of the genes related to neuron projection in Figure 1a and b. The transcription factors whose binding motifs were over-represented in the listed genes were co-analysed with their expression changes during the induction (Supplementary Table S7). Sox2, whose expression during the conversion lies between MEFs and primary neurons (Supplementary Figure S3A), was identified as the most likely candidate, because of the over-representation of its binding motif and the significant expression change during the conversion (Supplementary Table S7). In addition, late MET, rather than early EMT, is more similar to neuronal conversion when considering EMT and metabolism changes (Supplementary Figure S3). Thus, as a MET inducer [12, 21], Sox2 may play critical roles during the conversion.

**Figure 4:**
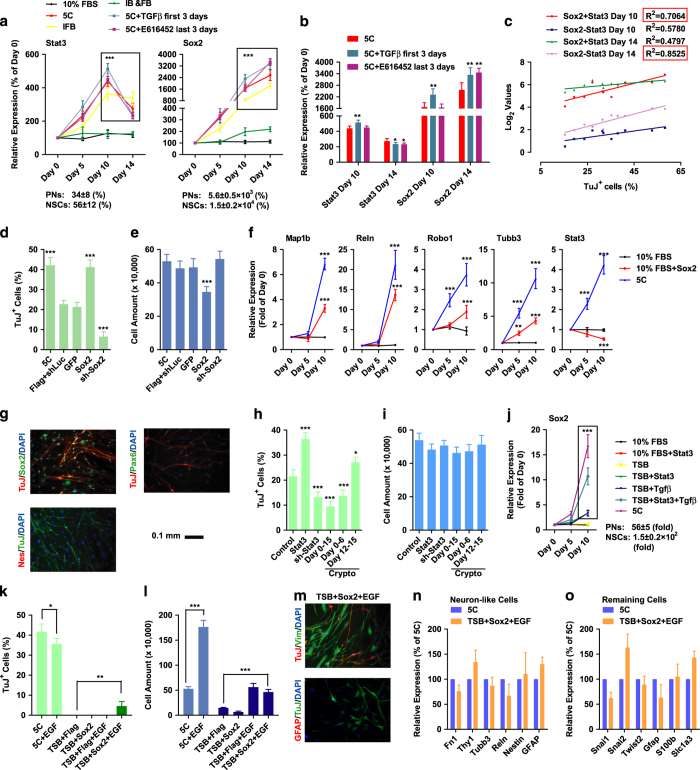
Sox2 replaces the sequential EMT-MET. (**a**) During neuronal conversion, insulin and bFGF regulated the expression of *Stat3* and *Sox2*, which could be modulated by TGFβ when used on days 0–3 and E616452 when used on days 12–15. The expression of *Stat3* and *Sox2* in primary neurons (PNs) and NSCs are also provided. (**b**) The expression of *Stat3* and *Sox2* on days 10 and 14 are listed. (**c**) The expression of *Stat3* and *Sox2* and TuJ^+^ percentages on day 16 that generated in Figure 3 were plotted against each other. The expression of *Stat3* and *Sox2* had higher correlation with the sum of the log_2_ increases of *Stat3* and *Sox2* on day 10 and the difference of these two log_2_ changes on day 14. (**d**–**g**) *Sox2* overexpression facilitated the induction of TuJ^+^ cells (**d**, **e**). *Sox2* overexpression up-regulated the expression of neuron projection genes rather than the NSC markers (**f**, **g**). (**h**, **i**) The abilities of *Stat3* and its inhibitor (Crypto) to regulate the current conversion were determined. (**j**) The abilities of *Stat3* to regulate *Sox2* expression were determined in different medium. The expression of *Stat3* and *Sox2* in PNs and NSCs are also provided. (**k**–**o**) Sox2 induced significant neuronal conversion with the help of EGF (**k**, **l**). The final cells were separated into two groups, neuron-like and remaining cells. The neuron-like were TuJ^+^ and the remaining cells were Vim^+^ but GFAP^−^ (**m**). These two types of cells were compared with 5C-induced cells were by quantitative PCR (qPCR; **n**, **o**).

The ability of insulin and bFGF to modulate the expression of *Sox2* and its upstream regulator during neuron fate specification, *Stat3 * [22], was then determined (Supplementary Table S6). Insulin, together with bFGF, induced *Stat3* up-regulation at the early stage (days 0–10) and down-regulation at the late stage (days 10–14; Figure 4a). *Sox2* expression was up-regulated by insulin and bFGF at both the early and late stage (Figure 4a). Multiple inhibitors like MK2206, U73122, Go6983 and LDN193189 were used to determine the mediator between insulin/bFGF and *Stat3/Sox2* (Supplementary Figure S7A–D). MK2206, an Akt inhibitor, attenuated the up-regulation of *Sox2* and *Stat3* and impaired the induction of TuJ^+^ cells (Supplementary Figure S7A–D), suggesting a pathway from insulin/bFGF to Akt and then to *Stat3/Sox2.*

As insulin and bFGF-induced neuronal conversion by accelerating cell proliferation and inducing sequential EMT-MET, the connection between sequential EMT-MET and up-regulated *Stat3/Sox2* was studied [23]. Actually, *Stat3* up-regulation was observed in more than 70% of EMT microarrays analysed previously (Supplementary Table S8 and [23]). In contrast to the universal up-regulation of *Stat3*, the up-regulation of *Sox2* and the four representative neuron projection markers were enriched in data sets with strongly induced EMT (Supplementary Table S8 and [23]). Therefore, the following hypothesis was proposed. Insulin- and bFGF-induced early EMT up-regulated *Stat3* and, subsequently, *Sox2* and neuron projection genes. When *Sox2* expression reached a certain criterion, its ability to induce MET overwhelmed insulin- and bFGF-induced EMT, leading to the switch from EMT to MET (sequential EMT-MET), the down-regulation of *Stat3*, and further conversion to neurons.

To confirm this hypothesis, TGFβ was used for the first 3 days, and E616452 was used for the last 3 days during the conversion. Both treatments facilitated neuronal conversion (Figure 3i–l), which correlated with enhanced *Sox2* up-regulation on both days 10 and 14, *Stat3* up-regulation on day 10 and *Stat3* down-regulation on day 14 (Figure 4a–c and Supplementary Table S6). Actually, by calculating the correlation between *Stat3/Sox2* expression and the percentage of TuJ^+^ cells, peaked up-regulation of *Stat3* on day 10 and the constant up-regulation of *Sox2* across whole conversion related highly to the high conversion efficiency.

Other evidences also support this hypothesis. First, the two strong EMT inducers, insulin and bFGF, are difficult to induce late MET (Supplementary Figure S3G and H). In addition, Sox2 bound to the locus of genes related to neuron projection in undifferentiated NSCs and resulted in their up-regulation during differentiation from NSCs to neurons, based on previous published RNA-seq and microarray results (Supplementary Table S9). Furthermore, *Sox2* induced MET in MEFs cultured with FBS medium, in NSC differentiation and during reprogramming (Supplementary Figure S7E–G and Supplementary Table S10). Actually, overexpression of *Sox2* during 5C-induced conversion impaired the early EMT and facilitated the late MET (Supplementary Table S6).

To further confirm the hypothesis above, expression of *Sox2* and *Stat3* was modulated during the conversion. Although retrovirus-mediated gene delivery impaired the conversion, *Sox2* increased the percentage of TuJ^+^ cells (Figure 4d and e). As *Sox2* was able to up-regulate the four representative neuron projection genes, *Map1b*, *Reln*, *Robo1* and *Tubb3*, in MEFs cultured with FBS medium (Figure 4f), an essential role for *Sox2* during 5C-induced conversion was suggested. In addition, *Sox2* promoted neuronal conversion without inducing the up-regulation of NSC markers, such as *Nestin* and *Pax6* (Figure 4g). *Sox2* overexpression decreased the percentages of both MEF-like and shrunken cells on day 14, but increased the percentage of neuron-like cells (Supplementary Figure S7H).

*Stat3* overexpression facilitated TuJ^+^ cell induction, while *Stat3* suppression with shRNA and cryptotanshinone (Crypto) inhibited this induction (Figure 4h and i). The ability of *Stat3* to induce *Sox2* expression was confirmed in TSB rather than FBS medium (Figure 4j).

Impairing the early EMT with TGFβ decreased the ability of 5C to induce the current conversion. However, *Sox2* overexpression attenuated the early EMT but promoted the conversion. In addition, considering the ability of early EMT to up-regulate *Sox2*, it is reasonable to suggest that the beneficial roles of early EMT during 5C-induced conversion can be attributed to *Sox2* up-regulation at least partially. Thus, *Sox2* may be able to replace sequential EMT-MET, especially the early EMT.

To confirm the hypothesis above, TSB medium was used since it was unable to induce sequential EMT-MET and *Sox2* up-regulation (Supplementary Table S6). Although *Sox2* was unable to induce TuJ^+^ cells in TSB medium, possibly because of the severe proliferation inhibition (Figure 4k, l and s Supplementary Figure S7G), *Sox2* induced low but significant levels of neuronal conversion with the help of epidermal growth factor (EGF; Figure 4k and l). The TuJ^+^ and remaining cells induced by *Sox2* in TSB+EGF were similar to those induced by 5C medium (Figure 4m–o).

Overexpression of *Sox2* in TSB medium with EGF induced MET directly instead of early EMT (Supplementary Table S6), further supporting the hypothesis that *Sox2* overexpression was a downstream effects of early EMT and led to further MET and conversion.

The ability of EGF to facilitate proliferation and to slightly inhibit TuJ^+^ cell induction suggested that sequential EMT-MET and proliferation were critical and sufficient for current conversion. The neuron-like cells generated with Sox2 and EGF were not significantly different from those generated with 5C medium when considering the expression of several fibroblast and neuron markers (Figure 4n).

Sequential EMT-MET is also observed during the generation of iPSCs when the four Yamanaka factors, Oct4, Klf4, c-Myc and Sox2, are introduced in a particular sequence: OK+M+S [12]. The 5C medium was approximately twofold more efficient than traditional mES medium in inducing GFP^+^ iPSC colonies with OKMS (Supplementary Figure S8A and B). Subsequent qPCR analysis suggested that 5C medium inhibited MET at the early stage but promoted it at the late stage (Supplementary Figure S8C and D). To validate this finding, Sox2 was removed from the reprogramming system, and an even larger difference in reprogramming efficiencies was observed, which correlated with enhanced up-regulation of *Stat3* and *Sox2* (Supplementary Figure S8E and F).

### Other neuron-induction processes

**Figure 5:**
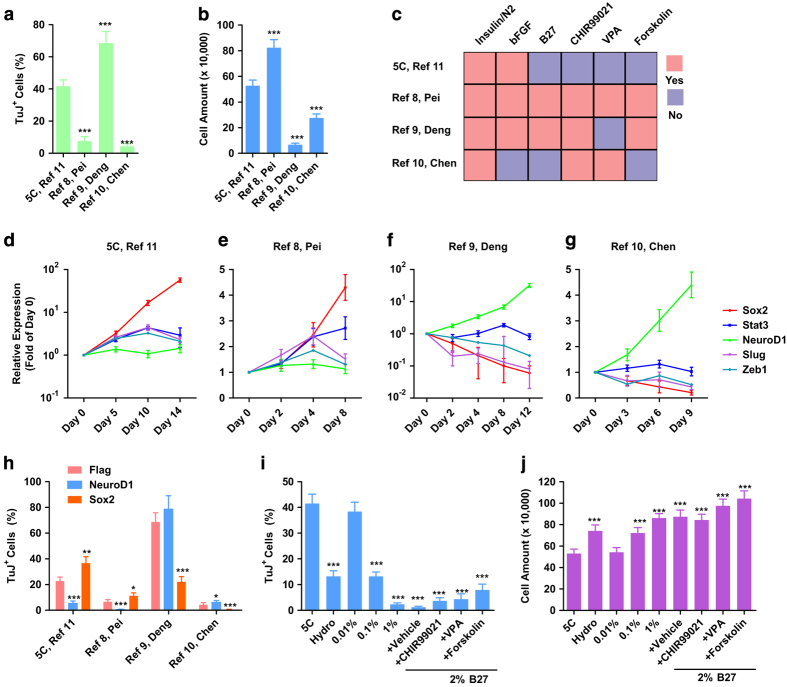
Neuronal conversion employs multiple pathways (**a**–**g**). Neuronal conversion was induced with 5C medium and other protocols from [ 8,9, 10]. TuJ^+^ cells were determined with FACS (**a**, **b**). The major components used in the induction phase of these protocols were compared (**c**). The expression of several representative genes was determined with qPCR (**d**–**g**). (**h**) The effects of *NeuroD1* and *Sox2* overexpression on different neuronal conversions. (**i**, **j**) The effects of hydrocortisone (hydro) and B27 on the current conversion were determined.

The other three reported protocols also induced TuJ^+^ cells from somatic cells [8,9,10]. We then applied these protocols in MEFs, and all three protocols successfully induced TuJ^+^ cells though with different efficiencies (Figure 5a–c). During the conversion induced by 5C medium or with the protocol provided by Pei Gang’s laboratory [8], up-regulation of *Stat3, Sox2* and EMT markers, such as *Zeb1* and *Slug,* was observed. However, *NeuroD1* up-regulation, which is considered a major mechanism for neuronal conversion [6, 7, 10], was not observed (Figure 5d and e). In contrast, the up-regulation of *NeuroD1* but not *Stat3*, *Sox2*, *Zeb1* or *Slug* was observed with the other two protocols (Figure 5f and g) [9, 10], suggesting that different routes may be employed during different neuronal conversions.

In addition, such hypothesis was supported by the different abilities of *NeuroD1* and *Sox2* to modulate different neuronal conversions. Overexpression of *NeuroD1* impaired the conversions with Sox2 overexpression, like 5C-induced or that reported by Pei’s Lab, while facilitated the other two conversions (Figure 5h). Overexpression of *Sox2* modulated the conversions just oppositely.

The major components of 5C medium, insulin and bFGF, were identified in four of the five conversion protocols, and the reason why the other protocols require additional compounds for the conversion might be the frequently used B27 (Figure 5c). A concentration of 0.1% B27 significantly inhibited the conversion (Figure 5i and j). Although the exact concentrations of B27 gradients are not available [24], hydrocortisone (hydro), which has been suggested to promote proliferation of primary glial cells and fibroblasts [25, 26], significantly inhibited neuronal conversion (Figure 5i and j). The other commonly used components, CHIR99021, VPA and Forskolin/cAMP, counteracted B27 and rescued the inhibitory effects of B27 to different extents (Figure 5i and j).

## Discussion

NeuroD1 is sufficient to convert astrocytes to glutamatergic neurons *in vivo* [6]. In addition, Isx9, which induces *NeuroD1* expression via Ca^2+^ influx, is involved in neuronal conversion [9]. However, *NeuroD1* overexpression significantly inhibited TuJ^+^ cell induction with the current 5C medium (Figure 5h and i). NeuroD1, a key regulator during adult neurogenesis, can be co-stained with several NSC markers, including Pax6, PSA-NCAM and DCX [27]. The undetected up-regulation of *NeuroD1*, *Pax6* and *Dcx* suggests that the conversion induced by 5C may represent a different way to acquire neuronal characteristics [11]. The ability of overexpressed *NeuroD1* to suppress *Sox2* further suggested the exclusivity of the conversions marked by *NeuroD1* or *Sox2* up-regulation [28].

The migration inhibition, proliferation arrest and metabolic switch suggest that the late or MET stage (days 10–14) of the conversion is substantially closer to a neuronal conversion than the early or EMT stage (days 0–10; Supplementary Figure S9). The function of the early EMT stage is to prepare cells for subsequent conversion. In addition, MET is a necessary early step during reprogramming from MEFs to iPSCs [29], and introducing a temporary EMT before MET further facilitates the process [12, 30, 31]. Thus, we have hypothesized that the early EMT may prepare cells to adopt a better intermediate state for further cell conversion processes [15, 16]. In the current study, Stat3 and Sox2 were identified as the downstream effectors of insulin- and bFGF-induced EMT. The low (around 150–200%) but universal up-regulation of *Stat3* during EMT supports its essential role. However, up-regulation of *Sox2*, *Map1b*, *Reln*, *Robo1* and *Tubb3* was observed only in samples with strongly induced EMT, suggesting the involvement of other downstream effectors of EMT (Supplementary Table S8).

As a MET inducer, *Sox2* serves as the regulator for the switch from EMT to MET; high levels of EMT activate *Sox2* expression and subsequently induce MET. Similar phenomena were also observed when we attempted to determine the optimal infection sequence of the four Yamanaka factors. *Sox2* can be delivered later than the other three factors, and the optimal sequence is OK+M+S [12]. This hypothesis was confirmed by performing the reprogramming with OKMS and OKM in 5C or mES medium (Supplementary Figure S8), suggesting that the 5C-induced, Sox2-related sequential EMT-MET also functions during iPSC generation.

## Materials and methods

### Animal studies

All procedures related to animal studies were performed in accordance with the National Institutes of Health Guide for the Care and Use of Laboratory Animals (NIH Publication No. 80-23) and were approved by the Institutional Review Board in Guangzhou Institutes of Biomedicine and Health. All efforts were made to minimize the number of animals used and their suffering.

Six- to eight-week-old CD-1 (ICR) male mice were housed in groups of three with free access to food and water. Mice were anesthetized by intraperitoneal injection with ketamine (90 mg kg^−1^) and xylazine (10 mg kg^−1^). Saline, 5C medium or IFB medium was then infused into the mouse brain (2.1 mm posterior to the bregma, lateral 1.2 and 3.2 mm to the skull) with an osmatic minipump (0.5 μl h^−1^, 14 days, Model 2002 and Brain Infusion Kit 2, Alzet, Cupertino, CA, USA). Each group had six mice, which was sufficient for the statistical analysis of preliminary studies. No particular method of randomization was used to assign the mice within each cage to the three groups, but no significant differences, especially in body weight, were observed. No mouse was excluded from the final analysis. On day 14, the connecting tube between the osmatic minipump and the brain infusion cannula was cut. The cannula was then sealed and kept in the mouse brain for an additional 2 weeks. On day 28, the mice were perfused with 4% paraformaldehyde after anesthesia and subjected to further analyses.

Mouse brains fixed with 4% paraformaldehyde were cut into 13-μm horizontal sections with a Leica EM FC7. Sections were prepared from 0.5 mm below to 2.0 mm above the end of brain infusion needle (3.7–1.2 mm to the skull). One in every twelve sections was analysed and used to re-construct the tissues inside and surrounding the needle track, to determine the volume of the tissue, to calculate GFAP intensity and to summarize the number of EdU^+^ cells.

### Neuronal conversion

Small-molecule compounds, growth factors and other cell culture materials used in current study are listed in Supplementary Table S1 with their final concentrations, catalog numbers and other necessary information required to repeat the current experiments.

MEFs were derived from 13.5-day ICR mouse embryos after removing the head and all internal organs. After recovery from freezing in liquid nitrogen, MEFs were cultured for two additional passages in non-coated plates with FBS medium. MEFs that normally attached strongly to the plate were further enriched. Thirty seconds of 0.25% trypsin treatment, followed by two gentle PBS washes, removed cells with neurogenic potential and weak attachment. Normally, 50 000 MEFs were plated per well after coating the 6-well plate with Matrigel for 0.5 h. The 5C medium or other induction medium was used for 14 days (live-cell tracing, qPCR and RNA-seq) or 16 days (FACS analysis and cell counting). Half of the medium was replaced with fresh medium every 2 days.

Retrovirus was produced with Plat-E cells and pMXs-based retroviral vectors. After adding polybrene to a concentration of 4 μg/ml, viral supernatant was used to infect MEFs twice, with a 24-h interval. 5C or other medium was used to replace viral supernatant 24 h after the second infection, and this time point was counted as hour 0 or day 0.

Primary neuron cultures were prepared as described previously [32]. In brief, the hippocampus and cortex isolated from mice within 1 day after birth were dissociated with neuron isolation enzyme. Cells were cultured for 3 weeks before maturation with neuron medium. Half of the medium was replaced with glial cell conditioned neuron medium every 2 days.

Primary NSCs were prepared from 13-day ICR mouse embryos [33]. In brief, after the meninges were stripped off, the remaining tissues of the brain were cut into pieces, rinsed with PBS and dissociated with 0.05% trypsin for 10–15 min. The dissociated cells were re-suspended in NSC medium. After the adherence of fibroblasts, the supernatant was cultured to form neurospheres. The NSCs were stored in liquid nitrogen after 5 additional passages to remove any possible contamination with other cells, such as fibroblasts.

As mentioned above, all primary cultures were prepared following previously published protocols. Marker gene expression was described in the manuscript. These cells were subjected to a mycoplasma test (MycoAlert Lonza, Allendale, NJ, USA) to ensure they were free of mycoplasma before use.

### Live-cell image tracing

Live-cell tracing was performed with a Cell Observer (Zeiss, Germany) during the current conversions. Light-field pictures were captured every 60 min. The time when FBS medium was replaced with 5C medium was recorded as hour 0.

Cell tracing was achieved with self-developed software and help from manual correction. In brief, individual cells were identified in each image with the location of the geometric gravity centers, the perimeter-to-area ratios and the length-to-width ratios. Doubling time, Td, was determined based on the log_2_ increase of the cell amount before and after a 24-h interval. The migration ability of one particular cell was determined as the distance between the cell on hour n and on hour n+1. If untraceable, the closest cell on hour n+1 was used for calculation. The following criteria were applied when cells were classified based on their morphologies. Neuron-like cells have a length-to-width ratio over 5 and at least two neurite outgrowths around the cell body. Shrunken cells have a small cell size, a nearly speherical shape (length-to-width ratio <1.5), and a high nucleus-to-cytosol ratio (over 1:5). MEF-like cells have both a large size and a low nucleus-to-cytosol ratio (below 1:10). Other cells were considered unclassified.

Live-cell tracing was performed with density of 20 000 MEFs per well, as it is difficult to trace cells at 50 000 cells per well or higher density. The Cell Observer normally captured two areas in each well of a six-well plate, which accounted for approximately one fourth of the well area. The self-developed software worked well on the first 7 days at 20 000 cells per well density. At the late stage, the software tended to identify several neuron-like cells located together as one single cell. Thus, cells were manually identified to correct the results from the software. In summary, counting total cell amounts, identifying and counting the three major types of cells, and calculating the migration distance were performed every 24 h during the conversion, with both the self-developed software and manual correction. Six independent replicates were analysed, each with ~5000 initial MEFs. The results are provided in Figures 1f–k and 2b–d.

Because the morphological changes and cell migration were strong during the conversion, the current self-developed software was only able to trace the cells before day 5. Thus, 500 initial MEFs from six representative replicates were selected and manually traced; the results are provided in Figure 2e–i and Supplementary Figure S4. Random selection was achieved by dividing each picture captured by the Cell Observer into 5×5 or 25 equal fields and selecting the cells that were closest to the middle of each field. The initial 500 selected MEFs from six independent replicates were traced. The final percentages of different types of cells were similar to those generated with software and manual correction from a large quantity of initial MEFs (at least six independent replicates each, with more than 5000 initial MEFs).

### Generation of mouse iPSCs

In brief, retroviruses encoding *Oct4*, *Klf4*, *c-Myc* and *Sox2* were produced with Plat-E cells and pMXs-based retroviral vectors. The viral supernatants were then used to infect MEFs (15 000 MEFs per well in a 12-well plate) with polybrene at 4 μg ml^−1^ [12]. The mES or 5C medium was used to replace the FBS medium on day 2 after two separate infections on days 0 and 1. Half of the medium was replaced with free mES medium every day. GFP^+^ colony counting was performed every 2 days from days 8–14.

### qPCR

Total RNA was extracted and reversed transcribed using the miScript system (QIAGEN, Valencia, CA), which also included a SYBR Green PCR kit following the manufacturer’s instruction. qPCR was performed on the CFX96 Touch Real-Time PCR Detection System (Bio-Rad). β-actin was used as the internal control to normalize the results from different samples. The primers used are listed in Supplementary Table S10.

### Immunofluorescence and FACS

The protocol followed those reported previously [11]. In brief, cells were fixed with 4% paraformaldehyde after removing the medium and washing three times with PBS. The 13-μm cryostat sections were prepared from mice perfused with 4% paraformaldehyde. These samples were washed twice with PBS and blocked with PBS containing 10% goat serum, 1% bovine serum albumin and 0.3% Triton X-100. Antibodies were diluted with PBS containing 1% goat serum, 1% bovine serum albumin and 0.3% Triton X-100 and incubated with the samples for 12 or 2 h at room temperature. Three PBS washes followed each antibody incubation. Immunofluorescence was detected with a Zeiss LSM810, and FACS assays were performed with a BD Accuri C6 flow cytometer (BD Biosciences, San Jose, CA, USA). Antibodies are listed in Supplementary Table S11.

TUNEL assays were performed with the ApopTag Fluorescein Direct *In Situ* Apoptosis Detection Kit (Millipore, Billerica, MA, USA) following the manufacturer’s instructions. Apoptotic cells were stained with red fluorescence and counterstained with DAPI. Apoptosis (%) was quantified by calculating the percentage of TUNEL^+^ cells in the total DAPI^+^ cells.

### Cell migration

The migration abilities of cells were determined by wound-healing and transwell assays. MEFs were subjected to different treatments such as 5C medium, trypsinized and replated at a density of 400 000 cells per well in a 6-well plate or in a transwell dish (3428, Corning, Inc., Corning, NY, USA). For transwell assays, cells that migrated out of the chamber were assessed 24 h later. For wound-healing assays, a 1-ml tip was used to create a wound 12 h after replating, and the cells that migrated into the wound area were assessed after an additional 12 h.

### EMT and other score calculations

Two methods were used to calculate EMT scores. The first method, which relied on the 130 genetic signatures of human EMT identified previously [34], was developed in our previous report [25]. In brief, the mouse genes homologous to the 130 human EMT signatures were identified based on information from Mouse Genome Informatics (MGI, http://informatics.jax.org/) and HUGO Gene Nomenclature Committee (HGNC, http://www.genenames.org/). These genes were divided into genes that were up-regulated and those that were down-regulated during EMT. The expression changes of these genes were summarized from RNA-seq or microarray data sets after comparing experimental groups to the control group. EMT score=(sum of log_2_ values of up-regulated gene expression changes−sum of log_2_ values of down-regulated gene expression changes)/overall number of genes used for calculation.

The second method was based on the qPCR results of four up-regulated genes, *Cdh2*, *Fn1*, *Slug*, and *Zeb1*, and two down-regulated genes, *Epcam* and *Ocln,* during EMT. The calculation method was similar.

The metabolism scores were calculated with the expression changes of genes in the oxidative phosphorylation (KEGG00190) and glycolysis pathways (KEGG00010). Metabolism score=(sum of log_2_ values of OX gene expression changes−sum of log_2_ values of Gly gene expression changes)/overall number of genes used for calculation.

### EMT microarray data sets

The 74 human and 31 mouse EMT microarray results have been previously collected and analysed [25]. In brief, searching ‘epithelial–mesenchymal transition’ or ‘EMT’ in GEO, removing the data sets that focused on primary cancer samples or tissues, and selecting the data sets for studying the EMT induced by TGFβ or Snai1/Slug/Twist overexpression resulted in 24 human and 17 mouse data sets. The biological replicates of the same treatment were averaged and further normalized to the control samples. If there were multiple treatments to induce EMT in one data set, the same control sample was used. The gene names in each data set were replaced with their official symbols in the downloaded annotations from MGI and HGNC. The data sets were merged based on the official symbols. The results were then transformed into log_2_ values. Finally, the data tables with 74 and 31 EMT-induction samples/pairs for human and mouse, respectively, were generated.

### RNA-seq and other data sets

RNA-seq data during induction were generated previously and deposited in the Gene Expression Omnibus under the accession number GSE68902 [11]. Gene ontology and KEGG analyses were performed with the DAVID online tool [35]. Pscan analysis was performed to identify potential transcription factor binding sites [20]. The other data sets downloaded from GEO are listed in Supplementary Table S12.

### Statistical methods

Experiments were repeated at least five times (*n*≥5) except for RNA-seq. No samples were excluded from the final analysis. The data met normal distributions, and comparisons were performed between groups with similar variance. The data were analysed and compared by two-tailed Student’s *t*-test, one-way analysis of variance with Dunnett’s *post hoc* test, or two-way analysis of variance with Bonferroni’s *post hoc* test using Prism 5 (GraphPad Software Inc., La Jolla, CA, USA). Error bars and ‘*n*’ represent s.d.’s (or standard errors if indicated) and the number of independent experiments, respectively. ‘*’, ‘**’ and ‘***’ represent significant differences (*P*<0.05), (*P*<0.01) and (*P*<0.001), respectively, versus the indicated control groups. The statistical information for each experiment or panel is listed in Supplementary Table S13.
