# Supplementary material for: Sequential EMT-MET induces neuronal conversion through Sox2
Source: Cell Discov. 2017 May 30;3:17017–. doi: 10.1038/celldisc.2017.17 (PMC5450022; doi:10.1038/celldisc.2017.17)
Supplement: Supplementary Information [file celldisc201717-s1.pdf]

Supplementary Information

Supplementary Figure 1

Serum removal counteracts 5C medium and impairs the conversion

(a-c) Cells cultured with 5C medium for 14 days were divided into two groups of cells, neuron-like and remaining cells. The expression of markers in fibroblasts, MEFs, primary neurons, astrocytes and NSCs were determined by qPCR (a-b). The results were further compared with the results generated in MEFs, primary neurons, astrocytes and NSCs in (c).

(d-h) MEFs were treated with serum-free medium for zero, one, two or three days, followed by a three-day culture with 5C medium. At the end of serum-free and 5C medium treatment, cell numbers were counted (n = 6, d), apoptosis was determined using a TUNEL assay (n = 6, e), and the expression of EMT/MET-related genes, including *Cdh2*, *Fn1*, *Slug*, *Zeb1*, *Epcam* and *Ocln,* was determined with qPCR (n = 5, f). The migration abilities of cells at the end of serum-free medium treatment were determined with transwell assays (n = 6, g). 5C medium was used to culture cells for an additional 13 days. Final cell amounts and percentages of TuJ^+ cells were determined on Day 16 (n = 6, h).

One-way ANOVA with Dunnett's post-hoc test was used.

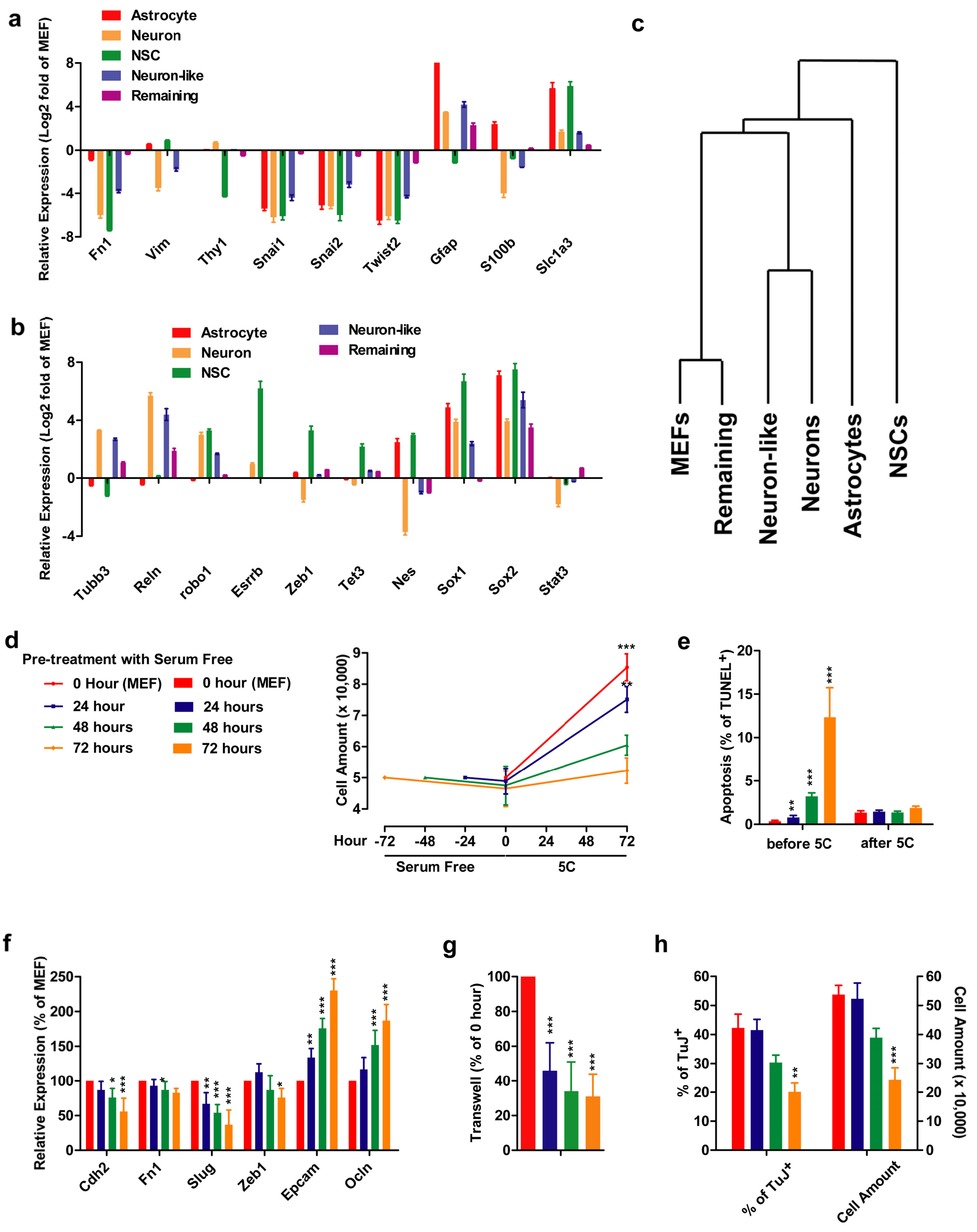
a
Astrocyte
Neuron
NSC
Neuron-like
Remaining
Relative Expression (Log2 fold of MEF)
8
4
0
-4
-8
Fn1
Vim
Thy1
Snai1
Snai2
Twist2
Gfap
S100b
Slc1a3
b
Astrocyte
Neuron
NSC
Neuron-like
Remaining
Relative Expression (Log2 fold of MEF)
8
4
0
-4
Tubb3
Reln
robo1
Esrrb
Zeb1
Tet3
Nes
Sox1
Sox2
Stat3
c
MEFs
Remaining
Neuron-like
Neurons
Astrocytes
NSCs
d
Pre-treatment with Serum Free
0 Hour (MEF)
24 hour
48 hours
72 hours
0 hour (MEF)
24 hours
48 hours
72 hours
Cell Amount (x 10,000)
9
8
7
6
5
4
Hour
-72
-48
-24
0
24
48
72
Serum Free
5C
***
**
e
Apoptosis (% of TUNEL+)
20
15
10
5
0
before 5C
after 5C
**
***
***
f
Relative Expression (% of MEF)
250
200
150
100
50
0
Cdh2
Fn1
Slug
Zeb1
Epcam
Ocln
g
Transwell (% of 0 hour)
100
80
60
40
20
0
***
***
***
h
% of TuJ+
Cell Amount (x 10,000)
60
50
40
30
20
10
0
% of TuJ+
Cell Amount
**
***

Supplementary Figure 2

Late stage of current conversion is closer to neuronal conversion

(a-c) Expression profiles of cells during 5C-induced conversion (Days 5, 10 and 14), MEFs, primary neurons, NSCs, ESCs, and iPSCs (passages 3 and 20) are listed in Supplemental Table 5. Gene expression in MEFs was used as control to calculate the $\log_2$ values for the other 8 samples. Although these samples were obtained from different GEO datasets, the $\log_2$ values of genes identified in all eight samples were further normalized to have similar averages and standard deviations. Cells with similar log-ratio expression profiles were grouped together by applying the Hierarchical Clustering method (a). EMT scores (b) and metabolism scores (c) were also calculated.

(d-f) RNA samples of cells during 5C-induced conversion (Days 5, 10 and 14), MEFs, primary neurons, primary NSCs, and R1 ESCs were collected. The expression of genes, including specific markers of these cells (d), EMT or MET markers (e), and genes regulating metabolism pathways (OX, Gly, f), was determined with qPCR ($n = 5$).

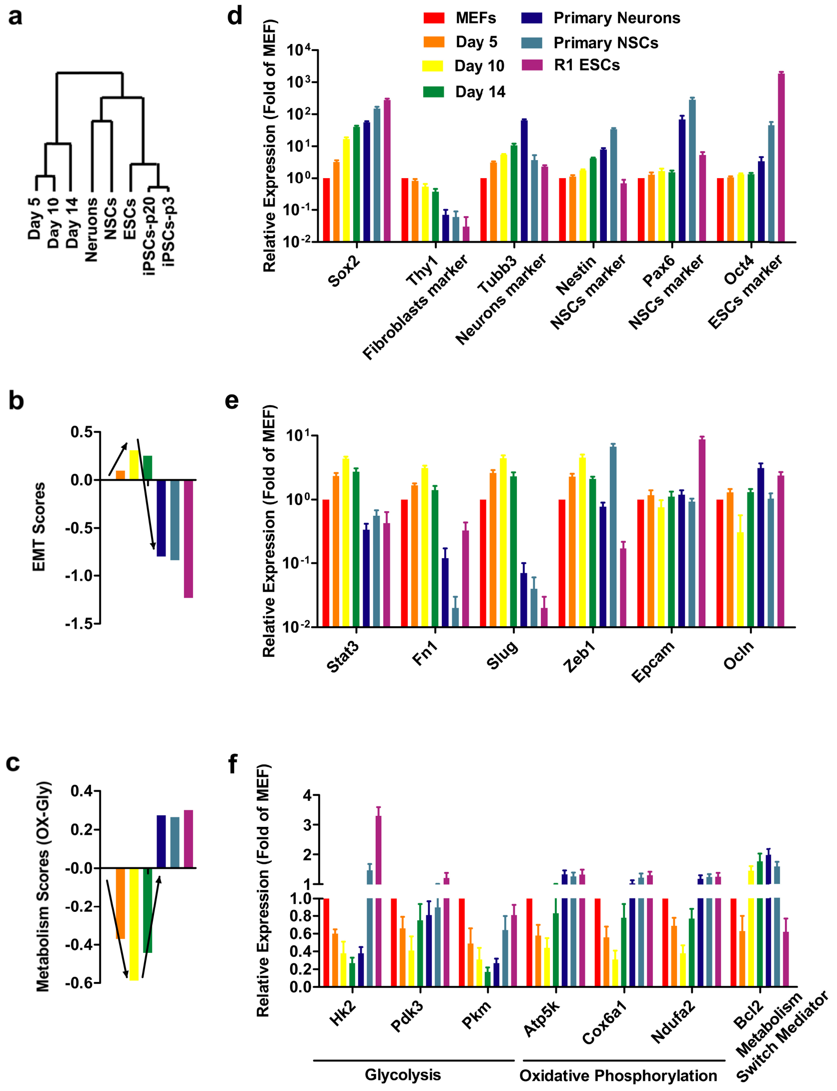

a
Day 5
Day 10
Day 14
Neruons
NSCs
ESCs
iPSCs-p20
iPSCs-p3
d
MEFs
Day 5
Day 10
Day 14
Primary Neurons
Primary NSCs
R1 ESCs
Relative Expression (Fold of MEF)
Sox2
Thy1
Fibroblasts marker
Tubb3
Neurons marker
Nestin
NSCs marker
Pax6
NSCs marker
Oct4
ESCs marker
b
EMT Scores
e
Relative Expression (Fold of MEF)
Stat3
Fn1
Slug
Zeb1
Epcam
Ocln
c
Metabolism Scores (OX-Gly)
f
Relative Expression (Fold of MEF)
Hk2
Pdk3
Pkm
Atp5k
Cox6a1
Ndufa2
Bcl2
Metabolism
Switch Mediator
Glycolysis
Oxidative Phosphorylation

Supplementary Figure 3

Induction of shrunken and MEF-like cells require mitosis

(a-c) Shrunken cells could be generated after a particular round of mitosis and continue to undergo mitosis, or via direct conversion (a). The contributions of the two routes to the final number of shrunken cells are summarized in (b). The average conversion times of cells that used these two routes are listed in (c).

(d-f) MEF-like cells could be generated with high mitosis or with low or even no mitosis (d). The contributions of the two routes to the final number of MEF-like cells on Day 14 are summarized in (e). The numbers of cells on Day 0 that used these two routes are listed in (f).

(g-i) The 500 initial MEFs were traced during the 5C-induced conversion. The numbers of neuron-like, shrunken and MEF-like cells that converted with high levels of mitosis are summarized across the conversion (g). The numbers of apoptotic cells during the generation of these three cell types are listed in (h) and normalized to the final numbers of these three types of cells (i).

Two-tailed Student's t-test was used for (c).

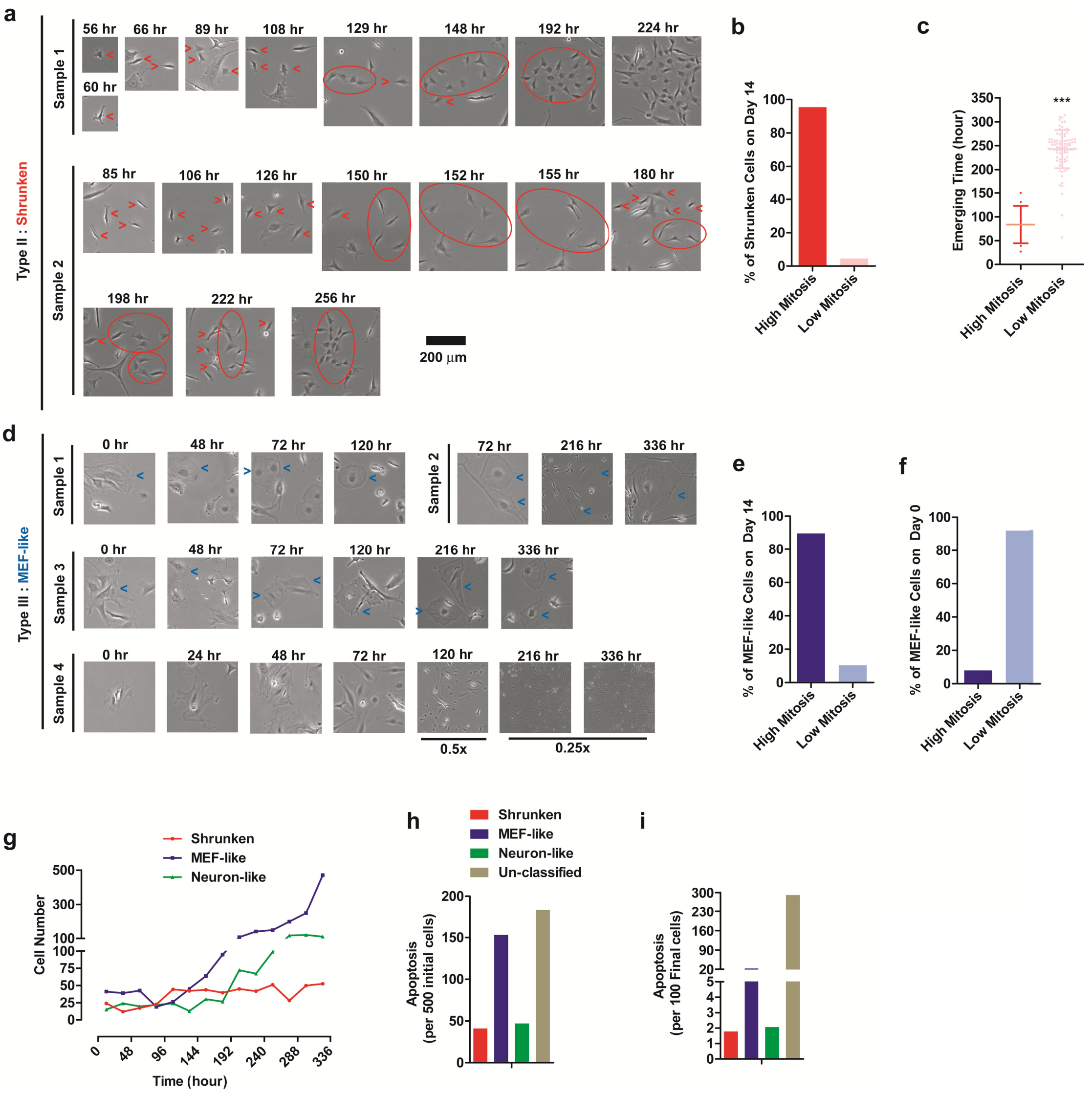
a
Type II : Shrunken
Sample 1
56 hr
60 hr
66 hr
89 hr
108 hr
129 hr
148 hr
192 hr
224 hr
Sample 2
85 hr
106 hr
126 hr
150 hr
152 hr
155 hr
180 hr
198 hr
222 hr
256 hr
200 µm
b
% of Shrunken Cells on Day 14
High Mitosis
Low Mitosis
c
Emerging Time (hour)
***
High Mitosis
Low Mitosis
d
Type III : MEF-like
Sample 1
0 hr
48 hr
72 hr
120 hr
Sample 2
72 hr
216 hr
336 hr
Sample 3
0 hr
48 hr
72 hr
120 hr
216 hr
336 hr
Sample 4
0 hr
24 hr
48 hr
72 hr
120 hr
216 hr
336 hr
0.5x
0.25x
e
% of MEF-like Cells on Day 14
High Mitosis
Low Mitosis
f
% of MEF-like Cells on Day 0
High Mitosis
Low Mitosis
g
Shrunken
MEF-like
Neuron-like
Cell Number
Time (hour)
h
Shrunken
MEF-like
Neuron-like
Un-classified
Apoptosis
(per 500 initial cells)
i
Apoptosis
(per 100 Final cells)

Supplementary Figure 4

High initial cell density activates the Notch pathway

(a) Log_2 values of expression changes of genes in Notch pathway during 5C-induced conversion are summarized in the heatmap.

(b) MEFs were converted by 5C medium at difference initial densities, 50,000 or 150,000 per well, in six-well plates. Notch pathway activation was demonstrated by NICD nucleus translocation on Day 10.

(c-d) Time-dependent effects of 5 μM Notch pathway inhibitor, DAPT, on proliferation and TuJ^+ cell induction are summarized. The effects of 50 ng/ml Jagged1, a Notch pathway activator, are also summarized ($n = 6$).

(e-f) DAPT differentially effected the proliferation of neuron-like cells and other cells, especially at the late stage ($n = 6$).

(g) The influence of initial MEF density on the final TuJ^+ percentages is summarized ($n = 6$).

One-way ANOVA with Dunnett's post-hoc test was used for (c-e and g), and two-way ANOVA with Bonferroni's post-hoc test was used for (f).

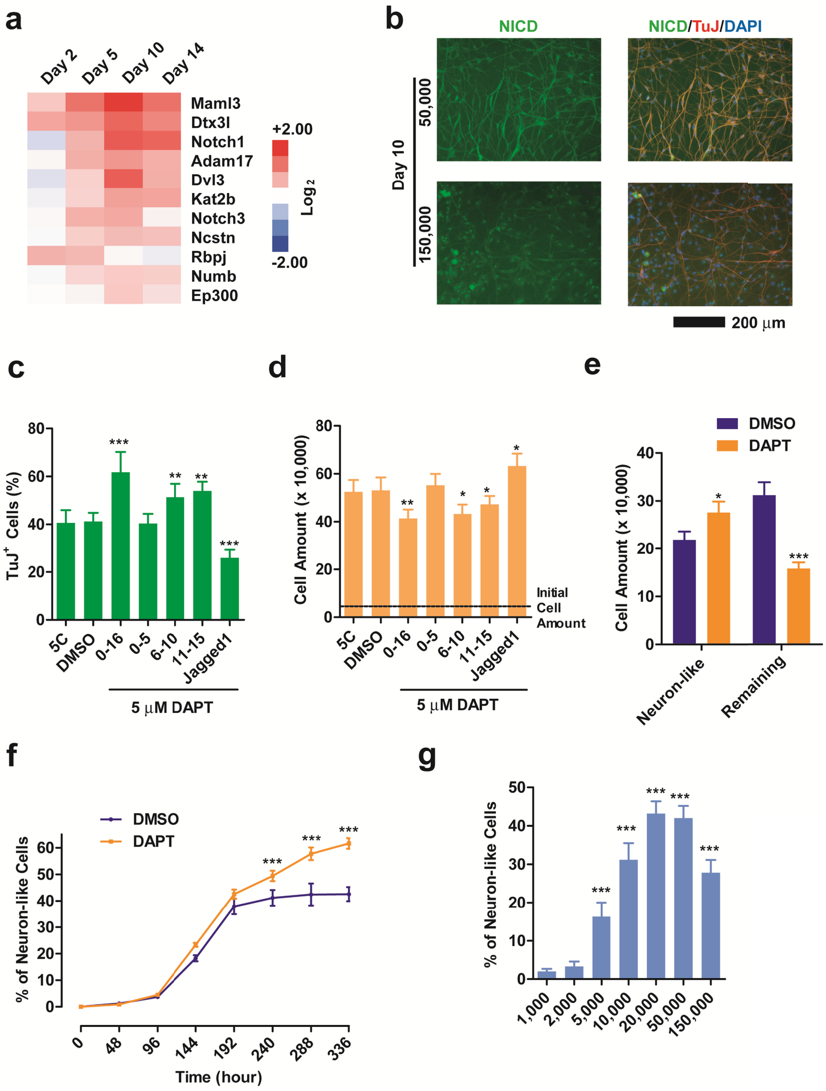
a
Day 2
Day 5
Day 10
Day 14
Maml3
Dtx3l
Notch1
Adam17
Dvl3
Kat2b
Notch3
Ncstn
Rbpj
Numb
Ep300
+2.00
Log₂
-2.00
b
NICD
NICD/TuJ/DAPI
Day 10
50,000
150,000
200 μm
c
TuJ+ Cells (%)
5C
DMSO
0-16
0-5
6-10
11-15
Jagged1
5 μM DAPT
d
Cell Amount (x 10,000)
Initial Cell Amount
5 μM DAPT
e
DMSO
DAPT
Cell Amount (x 10,000)
Neuron-like
Remaining
f
DMSO
DAPT
% of Neuron-like Cells
Time (hour)
g
% of Neuron-like Cells
1,000
2,000
5,000
10,000
20,000
50,000
150,000

Supplementary Figure 5

Insulin and bFGF induce cell migration

Wound-healing assays were used to determine the migration abilities of cells under the indicated treatments. Representative images from six independent experiments are listed.

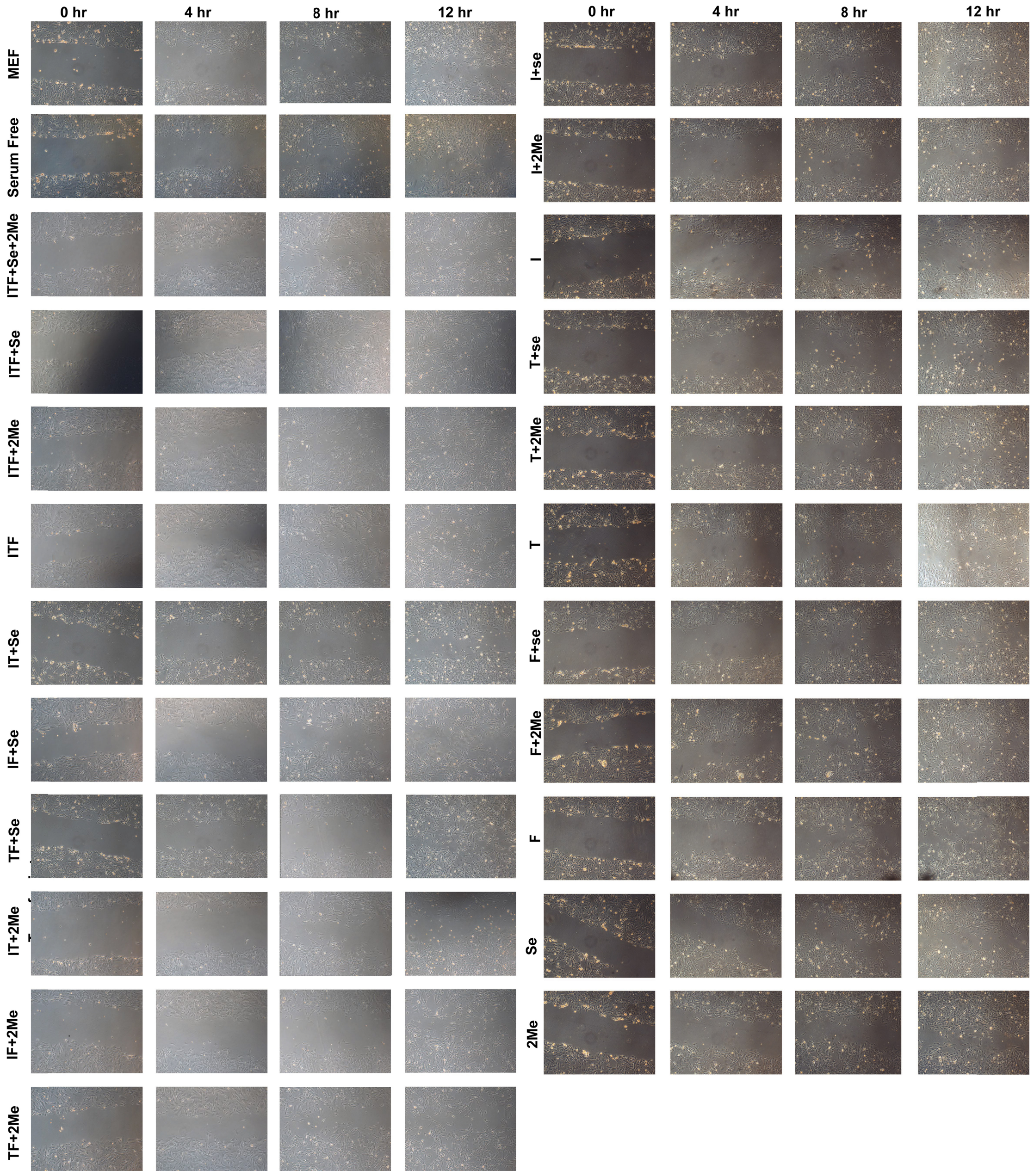

Supplementary Figure 6

IFB medium induces conversion in mouse brains

Eighteen mice were infused with 5C medium, saline, or IFB medium for two weeks (each group contained six mice). Brain sections were analysed after an additional two weeks.

(a) Schematic illustration of the brain infusion site and the recovery of the infusion tracks after the infusion with three types of medium.

(b) Representative images close to the end of the infusion track.

(c-e) Slides along the infusion track were analysed and used to re-construct the tissue inside and surrounding the infusion track. Volumes of recovered tissue (c), GFAP immunofluorescence intensity, and EdU$^+$ cells are summarized. The n and error bars were 6 and for s.e.m., respectively. One-way ANOVA with Dunnett's post-hoc test was used.

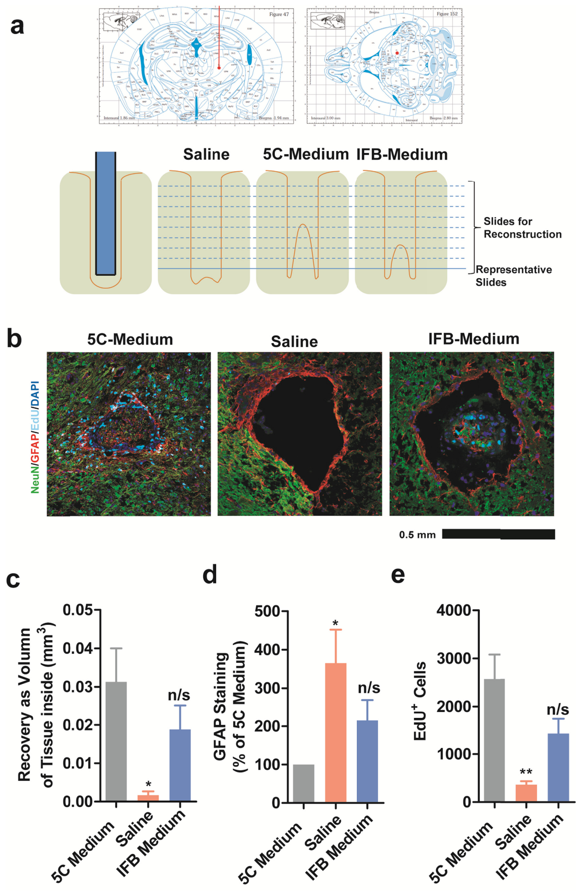

Supplementary Figure 7

***Sox2* inhibited cell proliferation**

(a-d) A total of 1 μM MK2206 was used during 5C-induced conversion. Expression of *Sox2* (n = 5, a) and *Stat3* (n = 5, b) was determined on Days 5, 10, and 14 with qPCR. The effects of 1 μM MK2206, 1 μM PD0325901, 0.5 μM U73122, 5 μM GO6983, and 0.25 μM LDN193189 on TuJ^+ cell induction and cell proliferation are summarized in (n = 6, c) and (n = 6, d).

(e) *Sox2* was overexpressed in MEFs, and the expression of genes related to EMT was determined with qPCR (n = 5).

(f-g) *Sox2* was overexpressed in MEFs. TGFβ and E616452 were also used. Cell migration (f) and cell cycle (g) were assayed on Day 3 with transwell assays and FACS (n = 6).

(h) *Sox2* overexpression increased the percentage of neuron-like cells but decreased the percentages of shrunken and MEF-like cells during the conversion.

One-way ANOVA with Dunnett's post-hoc test was used for (c-f), and two-way ANOVA with Bonferroni's post-hoc test was used for (a-b).

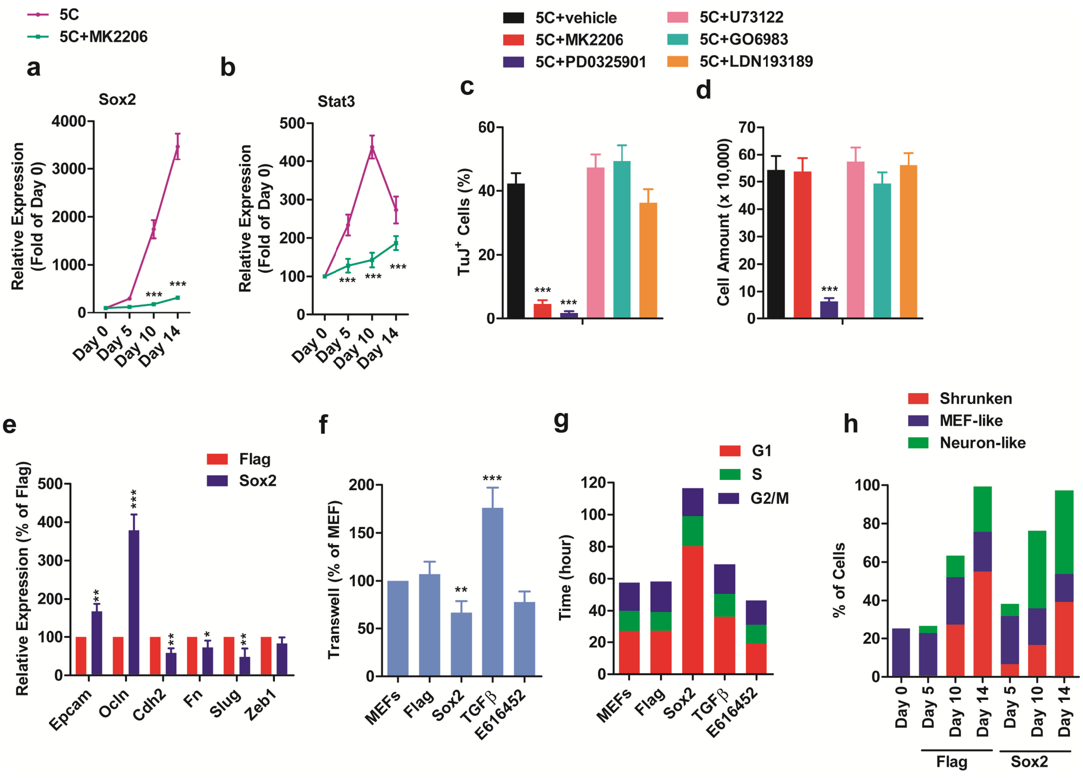

5C
5C+MK2206
a
Sox2
Relative Expression (Fold of Day 0)
Day 0
Day 5
Day 10
Day 14
b
Stat3
5C+vehicle
5C+MK2206
5C+PD0325901
5C+U73122
5C+GO6983
5C+LDN193189
c
TuJ+ Cells (%)
d
Cell Amount (x 10,000)
e
Relative Expression (% of Flag)
Flag
Sox2
Epcam
Ocln
Cdh2
Fn
Slug
Zeb1
f
Transwell (% of MEF)
MEFs
TGFβ
E616452
g
Time (hour)
G1
S
G2/M
h
Shrunken
MEF-like
Neuron-like
% of Cells

Supplementary Figure 8

Sox2 replaces sequential EMT-MET during reprogramming

(a-d) MEFs were reprogrammed by *Oct4*, *Klf4*, *c-Myc*, and *Sox2* with 5C or mES medium. GFP^+ colonies (n = 5, a) and cell numbers (n = 5, b) were counted during the reprogramming. Expression of genes related to EMT was determined on Day 3 (n = 5, c) and Day 8 (n = 5, d).

(e-f) MEFs were reprogrammed by *Oct4*, *Klf4*, and *c-Myc* with 5C or mES medium. GFP^+ colonies (e) were counted during the reprogramming (n = 5). Expression of *Stat*3 and *Sox2* was determined on Day 3 and Day 8 (n = 5, f).

Two-tailed Student's t-test was used for (c-d, and f), and two-way ANOVA with Bonferroni's post-hoc test was used for (a and e).

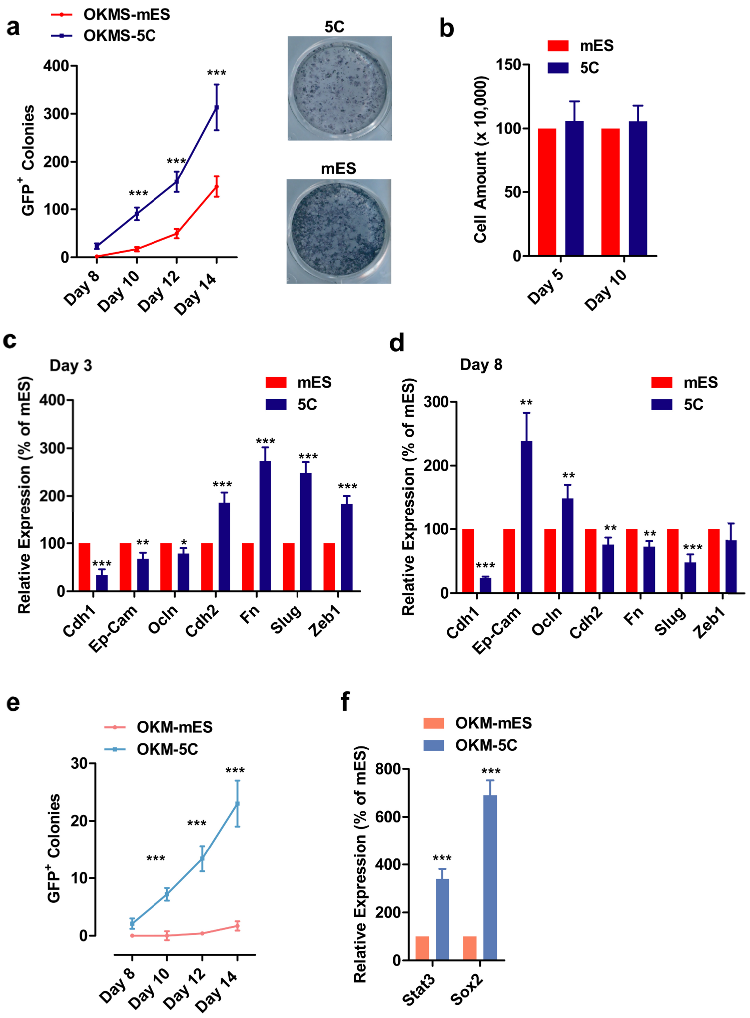

Supplementary Figure 9

Schematic illustration of sequential EMT-MET

bFGF and insulin in 5C medium support neuronal conversion by facilitating cell proliferation. In addition, bFGF- and insulin-induced EMT at the early stage results in *Stat3* up-regulation and subsequent up-regulation of *Sox2* and neuron projection. When *Sox2* expression reaches a certain criterion, a switch from early EMT to late MET is induced, which suppresses *Stat3* and further promotes neuronal conversion.

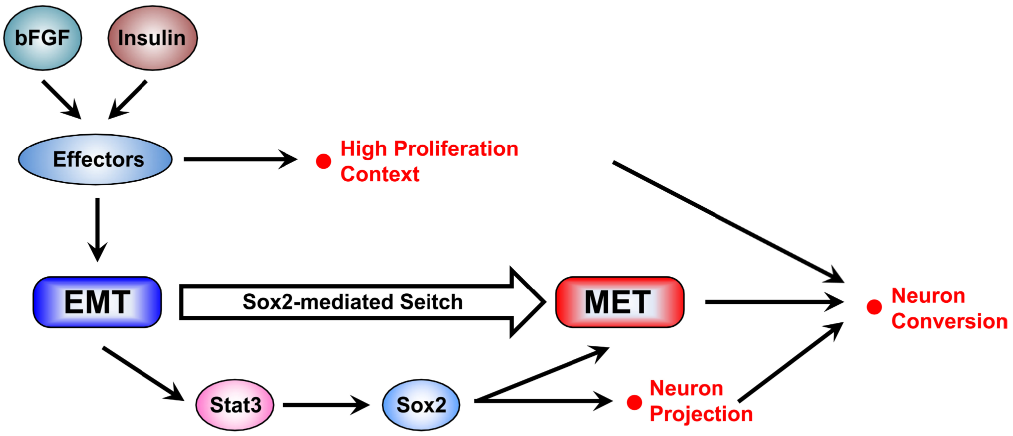

Supplementary Tables Legend

Supplementary Table 1

Medium and Materials used in the current research

Materials like growth factors and small-molecule compounds used in the current research were listed with their catalog numbers, purchased companies, and conventional final concentrations. Different combinations of these materials or medium composition were also listed in this Table.

Supplementary Table 2

Genes with significant expression changes were enriched in seven GO clusters

Gene expression profiles during the 5C-induced conversion was determined with RNA-seq. Genes with significant expression changes (over 2 fold) in at least one of the four time points were subjected for GO analysis and seven clusters of GO terms were identified. The genes in these seven clusters and their expression changes were listed. The average of $\log_2$ values of expression change of genes in different clusters were also calculated.

Supplementary Table 3

EMT scores calculated basing on RNA-seq results

EMT scores were calculated with two different methods as described in Methods. Briefly, gene expression changes of 130 genes which have been identified as EMT generic signature from RNA-seq data were used. The log2 values of expression changes of genes which normally are up-regulated during EMT were used as positive score, while those of gene that are normally down-regulated during EMT were used as negative score.

Supplementary Table 4

EMT scores calculated based on qPCR results

EMT scores were calculated with two different methods as described in Methods. Briefly, Gene expression changes of *Cdh2*, *Fn1*, *Slug*, *Zeb1*, *Ep-cam* and *Ocln* form qPCR were used to calculate another set of score. The log2 values of expression changes of genes which normally are up-regulated during EMT were used as positive score, while those of gene that are normally down-regulated during EMT were used as negative score.

Supplementary Table 5

Expression analysis from three GEO datasets for ESCs, NSCs, PNs, MEFs and conversion intermediates

Expression profiles of cells during 5C-induced conversion (Day 5, 10 and 14), MEFs, primary neurons, NSCs, ESCs, and iPSCs (passage 3 and 20) were downloaded from GEO database. Gene expression in MEFs were used as control to calculate the $\log_2$ values for the other 8 samples. Although these samples were obtained from different GEO datasets, the $\log_2$ values of genes identified in all eight samples were further normalized in order to have similar average and standard deviation.

Supplementary Table 6

qPCR results in cell cultured with 5C and other medium

MEFs were treated with 5C, 5C', IFB, FB, or IB medium for 5, 10 or 14 days. TGF β and E6161452 were used in time period as indicated. Expression of *Cdh2*, *Fn1*, *Slug*, *Zeb1*, *Ep-cam*, *Ocln*, *Sox2*, *Stat3*, *Map1b*, *Reln*, *Robo1* and *Tubbs* were determined

with qPCR. Three types of scores, EMT, dual-S and neuron scores were calculated from $\log_2$ values of expression change compared to MEFs.

Supplementary Table 7

Enriched binging sites of transcriptional factors via Pscan

Neuron projection genes identified in Fig 1a were used to find the enriched binding sites of transcriptional factors with Pscan. The transcriptional factors with enriched binding sites were used for further analysis. Final scores were calculated by multiplying the binding sites enrichment p-values ($-\log_{10}$) with the folds of maximum change during 5C-induced conversion.

Supplementary Table 8

Expression changes of Sox2 and other genes in different EMT datasets.

The 74 human and 31 mouse EMT microarray results were integrated as described in Methods. The results on *Sox2* and *Stat3* expression were extracted and subjected for further analysis. The totally 105 microarrays were classified into three groups with strong, medium, and weak EMT. The expression of *Sox2* and *Stat3* were summarized with in microarrays of these three groups.

Supplementary Table 9

Sox2 binding sites in NSC are close to neuron projection genes

Sox2 binging peaks were downloaded from GSE33024. The genes which are closest to the peaks were subjected for GO analysis and neuron projection (GO0048812/0031175) was enriched. The expression changes of these neuron projection genes during the differentiation from NSCs to Neurons were also listed

(from GSE24131, Supplementary Table 10).

Supplementary Table 10

EMT scores during NSCs differentiation and reprogramming with OK+M+S

Expression profiles during the differentiation from NSCs to Neurons (GSE24131) and during the reprogramming with sequential introduction of Yamanaka factors, OK+M+S (GSE39260) were downloaded from GEO database. EMT scores were calculated as described in Methods.

Supplementary Table 11

Antibodies and qPCR primers used in current research

As title.

Supplementary Table 12

Datasets used in current study

As title.

Supplementary Table 13

Statistics Information of each figure and table

As title.

Supplementary Video Legend

Supplementary Video 1

The generation of neuron-like cells during 5C-induced conversion.

Supplementary Video 2

The generation of shrunken cells during 5C-induced conversion.

Supplementary Video 3

The generation of MEF-like cells during 5C-induced conversion.
